# Essay on the Classification of the Insane

**Published:** 1839

**Authors:** 


### ESSAY ON THE CLASSIFICATION OF THE INSANE. By *M. Allen*, M.D. &c. London, pp. 212.

### ALLEN *versus* DUTTON. London, pp. 146.

THE first position assumed by Dr Allen is that "two establishments on the same grounds, should be allowed and encouraged for the purpose of classification;" and, growing out of it, his second is, that not only should there be two establishments on the same grounds, but these two should be sufficiently separated to prevent annoyance. These again he would have further divided into a front and back part, and the front so contrived as to be in appearance distinct from the back. And, in many cases, he considers an entirely separate house to be needed.

"I have known cases, the cure of which would have been apparently blighted and blasted for ever, if they could not have been wholly removed from, not merely the real, but the apparent, association of former scenes and circumstances, and this without any change in their servants and medical treatment and attendance, which is also essential." 4.

Truth, justice, and kindness constitute the basis proposed by Dr. Allen of all dealings with the insane. By the first, their confidence is secured; by the second, their judgment; and by the third their affections. Not more than about 3 per cent. of his patients suffer any personal restraint. And in the separate house under his own supervision he speaks of parties in every front division "who would, in their conduct and pursuits, and social enjoyments, put to shame many families who are reckoned perfectly sane. We have visiting parties from house to house, with the usual amusements of cards, chess, billiards, cricket, &c. For some months we published a weekly newspaper of considerable interest. Nor is it unworthy of notice, that some articles of a very superior kind in our critical journals have been written in this place; all which gives it more an air of social enjoyment and comfort, than the coldness and repulsiveness usually attendant on the loss of liberty, and forms within ourselves a little world of interest, better suited, I believe, to the state of the inhabitants than the real world could be to them."

Upon such a foundation, he goes on to rear his superstructure for the classification of the insane, and the first maxim laid down is, that to *tranquillize the mind*, is the best preparation for restoring the affections to their equilibrium, and the natural functions of the body to their healthy state; for dispelling the mists of delusion, and bringing back to its vacated throne, the fugitive understanding.

Another and scarcely less important principle is insisted upon—namely, the treatment of the insane being delicate and intellectual. The author cites several interesting cases of mental derangement, where the apparently hopeless condition of the patient was at first ameliorated and finally changed to a condition of soundness and healthfulness—and we can only regret that our limits do not allow of our citing them.

Dr. Allen lays it down as another essential principle to know every extreme view and error to which the human mind is liable, "and where these exist, as in most cases of insanity, to endeavour to counteract them by clear and beautiful views of the truth."

"What is of the greatest consequence is, that it is still more necessary to know the best mode of making truth admissible and effectual; for it ought never to be forgotten, that in all cases where error and delusion exist, even if we know those views which are best calculated to counteract and remove them, still more depends on the manner, circumstances, and spirit in which we present and apply them." 46.

To establish, cultivate, and encourage in lunatics the habits of self-restraint, and self control, to induce them to restrain themselves, to control their own actions, in preference to making them feel the restraining and controlling power of another, is also a great principle, though not, perhaps, sufficiently carried out. In the eloquent language of our author, whose words are always preferable to our own:—

"NOTHING BUT ABSOLUTE NECESSITY SHOULD JUSTIFY ABSOLUTE RESTRAINT. *It must always be considered as an evil to which we are reduced, in order to avoid a greater.* Whenever it is unnecessary and continued too long, it will do more harm than good: the furious will be made more furious, and the suicide more determined to effect his purpose. Whenever the patient is indulged with more liberty and behaves better, we must have forbearance to the utmost extent, and submit to all possible risks, losses, and expenses, rather than again have recourse to it; and when it is repeated, the patient must be made, if possible, to feel that it is deserved.

Their faults, like those of children, must be viewed with pity. They are the wild displays of feeling, without understanding. We must make excuses for them, often overlook, as often visit them slightly, only seldom with seriousness, and always with moderation, justice, and prudence. No evil is greater than the evil of constantly chiding and suspiciously looking for faults. It is an evil spirit that poisons and inflames everything within its sphere. A contrary spirit has a healing influence; though it requires numerous attendants, and makes the whole business of superintending the insane a source of constant and intense anxiety and solicitude." 68.

But these may rather be said to be principles of treatment, than rules for classification. Some of these we now proceed to notice.

Some cases require to be associated with others of an opposite character, grave with gay, and lively with severe; for such, by being thrown together, neutralize, like acids and alkalies, each other.

Other cases, on the contrary, require assimilation, in order to awaken comparison, and are cured by the patient witnessing his own condition caricatured in that of his neighbour.

Many cases, again, in their incipient stages, while requiring seclusion and separation from friends, would be only aggravated were the sufferers to be set down at once among other patients.

The necessity is obvious of separating the boisterous, dirty, and epileptic, from the more rational, the cleanly, and the nervous.

The prevention of bad habits in some, by the constant presence of others, so as to awaken the sense of shame, and the habit of self-restraint, calls for another division of patients. This requires great tact and discrimination in the association. The preferable class must not be injured by their intimacy with the more degraded, while the object of improving the latter by their society, is sought.

In proof of the practicability of conceding with safety, much real, or apparent liberty, Dr. Allen adduces, in opposition to the common dread of suicidal attempts, the fact, that in his own experience, he has had but one occur of suicide out of 600 patients, and that person "had secreted a rope and effected his purpose the day after his arrival, by pretending to retire to a place of convenience."

Another fact, certainly not less striking, will serve to show the safety with which many patients might be allowed greater freedom of locomotion, even so far as their personal detention at a particular residence is concerned.

"It is remarkable," we give the statement in the doctor's own words, "that many have in an incipient state of convalescence, been placed on parole of honor: first, they are simply restricted to the garden, and afterwards to the fields; and if no breach of confidence occurs, they are allowed a pass key to go out and in when they please, and scarcely an instance has occurred in which they have taken advantage of this privilege, to make their escape; nor have they opened their doors to others. *Those who escape are always those who are not so trusted.*" 98.

The "Essay on Classification" forms but the smaller third of the entire volume, another third is taken up with an appendix of cases, one of which we shall transfer to our own pages. And the remainder of the book is occupied with a report of the case of Allen versus Dutton, in which, it being a matter altogether personal, our readers would feel but little interest. The plaintiff obtained a verdict—and appears also, upon motion for a new trial, to have received the opinion of the Judges on his behalf through the goodness of his cause.

The only case for which we have left ourselves space, is one of an encouraging character, and one which, as Dr. Allen confesses that to it he is indebted for much of his success, we have some pleasure in selecting.

The patient was a dissenting minister, of partial and gloomy views, pushed, of course, into extremes. A zealot, violent and vindictive. Every opposite quality of his mind was in excess, and had always been in a highly excited and irregular state. He had been for many years kept in a painful and irritable condition; and conjugal infelicity with domestic strife had just before terminated in mutual separation. The form of his derangement was thus easily traceable to the circumstances by which it had been induced.

"He was in a state of the most furious mania;—his was one of the most violent and distressing cases I had ever seen. It is impossible to convey any adequate conception of its appalling nature. His language was obscene and vulgar, and his horrible oaths and blasphemous speeches were poured forth for some weeks without ceasing, and without sleep, with a volubility, rapidity, and a voice so loud, and so foaming with passion, and with such a frightful expression of countenance, that even those most accustomed to such scenes, and of the strongest nerves, trembled before him. He had a demoniac energy and eloquence, which was, indeed, of the most harrowing awful kind. It was truly terrific! for even at a distance, his voice sounded like a river escaping from some narrow part, and rushing with impetuosity over every thing that would impede its course.

One principal subject of his furious raving, was his favourite doctrine of election; or rather, perhaps, I ought to say, his blasphemous doctrine of reprobation. He was constantly denouncing every one (and against myself he was peculiarly severe) as lost, whose belief on this point was not, even in phraseology, the same as his own; calling on God to execute vengeance upon them; then blaspheming God, that his prayers and commands were neither heard nor obeyed; taunting and cursing him with a contempt which no language can describe; calling his clemency weakness, and his not executing his decrees a proof he did not possess the power he pretended to have.

On the subsidence of his excitement, he was overwhelmed with the perfect recollection of all he had uttered during the utmost fury of his dreadful ravings, and his state was truly miserable and deplorable. In this state I took great pains with him, treated him with every possible kindness, and endeavoured to shew him every possible mark of my confidence, one instance of which may be mentioned. I gave him, at a very early period of his convalescence, a set of manuscript sermons, all in loose slips, and which he read with great pleasure, and preserved with great care. * * * The spirit of these discourses just suited his altered state of mind, for he himself felt horror-struck at the views which had led to such awful consequences.

Though his furious state was so unusually violent, yet it was of long duration, and after it had left him, it was some time before he was able to overcome the painful reflections which came over him; he, however, recovered, and returned home in the September following, since which period I have received many, and almost constant proofs of his great gratitude and attachment to me."

With this case we close our notice of Dr. Allen's work, by recommending it to the perusal of the reading public, particularly of those employed in the custody of insane persons. We cannot do so, however, without protesting against the slovenly hotch-potch shape in which it has been transmitted to us. It has a title, it is true—but there is no date to the title-page—and even Dr. Allen appears to have his part of the blame, for the Essay, is not what it purports to be, an Essay "on Classification," but rather on the moral as opposed to the mechanical treatment of insanity. And having been commenced as part of a personal defence against his already overthrown adversary Mr. Dutton, although the defensive attitude is soon abandoned, there is an incongruity and total want of connectedness about the Essay itself. Indeed, the whole work is a misnomer—the numerous cases in the appendix, have nothing whatever to do with the subject of classification, from beginning to end—and, as little to do with it has the disgusting history of the poor woman whose case was brought into court on the trial, to which we have already assigned our reason for not inviting more particular attention.

